# Trace element distribution in the snow cover from an urban area in central Poland

**DOI:** 10.1007/s10661-015-4446-1

**Published:** 2015-04-03

**Authors:** Patrycja Siudek, Marcin Frankowski, Jerzy Siepak

**Affiliations:** Department of Water and Soil Analysis, Faculty of Chemistry, Adam Mickiewicz University in Poznań, Umultowska 89b Street, 61-614 Poznań, Poland

**Keywords:** Trace metal, Environmental monitoring, Snowfall, Coal combustion, GF-AAS analysis, Long-range transport

## Abstract

This work presents the first results from winter field campaigns focusing on trace metals and metalloid chemistry in the snow cover from an urbanized region in central Poland. Samples were collected between January and March 2013 and trace element concentrations were determined using GF-AAS. A large inter-seasonal variability depending on anthropogenic emission, depositional processes, and meteorological conditions was observed. The highest concentration (in μg L^−1^) was reported for Pb (34.90), followed by Ni (31.37), Zn (31.00), Cu (13.71), Cr (2.36), As (1.58), and Cd (0.25). In addition, several major anthropogenic sources were identified based on principal component analysis (PCA), among which the most significant was the activity of industry and coal combustion for residential heating. It was stated that elevated concentrations of some trace metals in snow samples were associated with frequent occurrence of south and southeast advection of highly polluted air masses toward the sampling site, suggesting a large impact of regional urban/industrial pollution plumes.

## Introduction

Snowpack is a good indicator of air pollutants, i.e., sulfate, nitrate, and trace metals. It is well established that accumulation and transformation of chemical species in a snow cover have an impact on groundwater/terrestrial environment and human health (Dossi et al. 2007; Engelhard et al. 2007). Many of these contaminants (e.g., heavy metals: Hg, Ni, Pb, Cd) are known as hazardous. For instance, mercury—a potent neurotoxin—can reinforce the negative effect on biotic and abiotic processes in ecosystems, especially those located near the emission sources (Wiener et al. 2003). Atmospheric processes such as dry and wet deposition of trace elements play a crucial role in cleansing mechanism. The major sources of trace metals (TMs) and metalloids are associated both with natural (rock weathering, mineralization, dust storm, volcanic eruption, e.g., Gabrielli et al. 2008) and industrial processes such as fossil (As, Cu, Co, Cr, V, Ni, Sb, Fe, Mn, Zn, Sn) and oil combustion (Mn, Pb, Fe, Ni), motor vehicle exhausts (Pb, Cu, Cr, Sn, Sb), smelting (Ni, Cu, As, Pb, Cd), iron/steel manufacturing (Cr, Mn, Ni, Co), waste incineration (Pb, Zn), and cement production (e.g., Pacyna and Pacyna 2001). A large number of industrial/urban activities result in significant spatial and temporal variability in TM concentration in the lower atmosphere (Melaku et al. 2008). Several studies have highlighted that atmospheric cycling of TMs (dispersion, transport, deposition) depends on a broad spectrum of environmental factors, i.e., physical-chemical properties of species, weather conditions, orography, and particle size distribution. Therefore, understanding of chemical processes in the snow cover and the relationships between TM concentration and meteorological variables seems to be a fundamental issue in determining the impact of local/regional anthropogenic sources.

Numerous empirical and modeling studies that focused on chemical composition of a snow cover have been intensively undertaken at polar sites; however, similar measurements in polluted European regions are still scarce. Since the urban environments are heavily affected by anthropogenic pollutants, a quantitative analysis of TMs in the snow cover system could be of crucial importance. Recent studies conducted in major urban and industrial centers showed higher contents of some TMs in a short-term snow cover at roadsides/crossroads as a consequence of exhaust emission from the traffic (Loranger et al. 1996; Engelhard et al. 2007; Vasić et al. 2012). Based on multi-year measurements of TM concentration, Moreno et al. (2011) found extremely high concentrations of metals in technogenic gasses and particles, suggesting increasing trend in anthropogenic emission during the cold season. Becagli et al. (2012) demonstrated that metals such as V, Ni, Fe, and Al could be present in particulate phase as free metal ions, carbonates, oxide hydrates, or labile complexes with organic ligands. Similar studies emphasized large seasonal variation in the concentration level of trace elements in a snow cover with pronounced differences between urban and non-urban sites. On the other hand, field-based studies provided an evidence for the large impact of transported pollutants on local and regional scale. Furthermore, some observations explained the relationships between TM dynamics in snowpack and pH range, changing weather conditions, particle size, and proximity to major anthropogenic sources (Melaku et al. 2008; Vasić et al. 2012). Followed by those findings, further studies seem to be highly desirable to explore reactions in the ecosystem and to estimate the potential of aquatic and soil system contamination after snow melting. Trace element concentration in mid-latitude snowpack is not well documented, and only several investigations have been previously performed (Bacardit and Camarero 2010). In this context, a series of winter measurements in the freshly fallen snow cover was conducted in Poznań, a medium-sized city in central Poland characterized by different industrial activities.

The main goal of this study was to determine concentrations of trace elements (Cd, Zn, Ni, Cu, Pb, As, Cr) in the shallow snow cover and identify meteorological factors that control their inter-seasonal variability. During the field campaigns from January to March 2013, the eight independent experiments were performed and a modern approach to snow sampling was proposed. Since the TM fate and behavior in the atmosphere is closely linked to different sources (local and regional), the effect of long-range transport from adjacent regions on metal distribution in the snow cover was also evaluated. Finally, the measured TM concentrations in snow were compared with experimental data from other urbanized regions in Europe and USA and cold environments including poles and high mountains.

## Materials and methods

### Study area

Poznań is a city (area of 261.8 km^2^, >600 000 inhabitants) in the Wielkopolska Province, central Poland. The study area is constantly influenced by various emission sources. The industrial and urban activities in this region are as follows: coal-burning heat and power plants, individual power generating stations, and domestic furnaces where coal is the main fossil fuel. Moreover, such sources as dumping grounds for municipal wastes, hospital and domestic sewage, cement factories, sewage treatment plants as well as different industrial units producing metals and paints, high-temperature industrial processes (smelting, waste incineration), heavy traffic, and agricultural activities also contribute to trace metal emission.

The annual mean temperature in Poznań is about 8.5 °C, and the prevailing wind direction is from the northwest. Poznań has the lowest yearly sum of precipitation in Poland (<550 mm) and 32 % of the fall occur during winter season. In the study area, the number of days with the snow cover and temperature below 0 °C is relatively low as compared with high mountains or poles. Snow typically covers the ground from November to March, and the length of that period is controlled by local meteorological conditions (city heat island influence) and the characteristics of NAO index (temperature/precipitation amount). Thus, the depth and occurrence of the snow cover may vary both spatially and temporally, even within a short period of time. In this study, the formation time of the shallow snow cover ranged from 10 to 24 h. Table 1 presents details of each snow event and meteorological background during the series of eight field experiments in Poznań.

**Table 1:** Statistical characteristics of atmospheric conditions in Poznań during the sampling days in winter 2013

Date	Experiment code	Mean temperature ± SD (min-max)	Mean pressure ± SD (min-max)	Mean wind speed ± SD (min-max)	Prevailing wind direction	Snow cover depth (cm)	Number of snow layers
25/01/13	J1	−6.6 ± 4.0 (−12.4 to −0.2)	1011 ± 1.2 (1008–1012)	0.2 ± 0.3 (0.1–1.1)	N, NE	15	11
28/01/13	J2	−2.9 ± 2.2 (−6 to −0.2)	1001 ± 3.4 (996–1005)	1 ± 0.3 (0.6–2)	NW, W	10	9
13/02/13	F1	1.1 ± 1.4 (−0.7 to 3.3)	1004 ± 0.9 (1002–1005)	0.7 ± 0.2 (0.5–1.4)	S, SE	6	5
14/02/13	F2	−0.4 ± 0.9 (−1.9 to 3.4)	1014 ± 1.9 (1011–1016)	0.3 ± 0.3 (0.2–1.1)	SE, E	10	10
19/02/13	F3	−0.5 ± 0.5 (−1.2 to 1.6)	1010 ± 2.9 (1004–1014)	1.4 ± 0.5 (0.6–2.2)	NW, W	12	11
11/03/13	M1	−2.9 ± 0.6 (−3.8 to −2.0)	995 ± 2.0 (991–997)	0.2 ± 0.7 (0.1–2.4)	NE, E, SE	20	13
19/03/13	M2	0.1 ± 3.1 (−4.7 to 4.5)	1006 ± 2.7 (1002–1010)	2.5 ± 0.7 (1.1–3.7)	S, SE	25	16
25/03/13	M3	−3.3 ± 5.9 (−13.7 to 2.2)	1016 ± 0.9 (1015–1018)	0.5 ± 0.4 (0.1–1.5)	NE, E	20	14

Additionally, the term “*heating season*” was used to highlight the special period during the whole calendar year when intensive domestic heating occurs. In our study, snow experiments were considered in three different periods, representing the month of the sampling duration in 2013. In particular, snow events were categorized into three types and described as follows: J—snow experiments performed in January (two case studies, labeled as J1 and J2), F—snow samples collected in February (case studies: F1, F2, and F3), and M—the snow cover sampled in March (case studies: M1, M2, M3).

### Sampling strategy and laboratory analyses

**Fig. 1:**
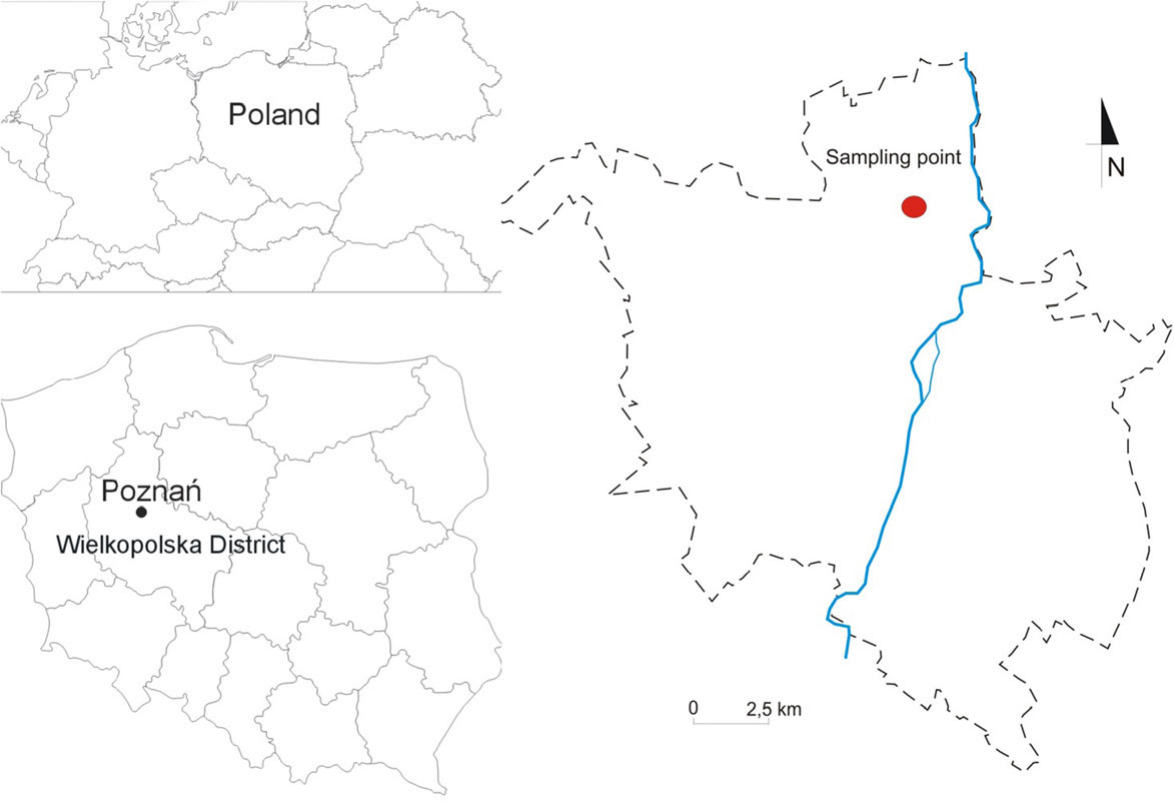
Map of the sampling site in the urban area, Poznań, Poland

Snow samples were collected at the urban site located in the main campus of Adam Mickiewicz University in Poznań (52° 42′ N, 16° 88′ E) during winter measurement campaign in 2013 (Fig. 1). The sampling site (POZ) represents urban area that is affected by different types of anthropogenic sources (fuel combustion, industrial processes, and minor role of heavy traffic). In particular, the area up to 30 km southeasterly from the sampling location is the most industrially impacted zone having a high contribution to the air pollution of the study site, whereas regions located to the north represent mainly rural and sub-urban areas. The majority of the point pollution sources is located within 5–10 km from the sampling site (i.e., CFFPs Poznań Karolin and metallurgical factory ∼5 km southeast of POZ, residential boilers, waste incinerations in Poznań, Biedrusko military area ∼10 km north of POZ, rail freight transport, and roads ∼1 km south of POZ).

All field measurements were performed at flat terrain and far from buildings in order to eliminate the influence of particles emitted during the wintertime heating from nearby buildings as well as the potential contamination from underlying soil via re-suspension. The sampling site was visited each time after the snowfall, and as a result, eight experiments were performed during the whole observation period. Plastic bags and gloves were used so as to avoid negative artifacts during snow sampling. The snow cover was collected through the use of a pre-cleaned Plexiglas device and a plate. Each snow column was in situ divided into several subparts (∼1, 2, and 3 cm layers, depending on the possibility to select min. 1-cm thick snow strata). The scheme was as follows: starting from the surface layer (snow-air interface), each subsample was carefully separated from the snow column, placed in a pre-cleaned polyethylene bag, and subsequently transported to the laboratory. Detailed description of the sampling scheme used during our snow experiments in 2013 can be found elsewhere (Siudek et al. 2014). Three depth-integrated snow columns were taken immediately after the snowfall in order to exclude the possible artifacts, i.e., atmospheric input from the deposition of dust or drifting/blowing snow. In this study, we did not calculate the detailed time of the snow cover occurrence; therefore, it was not possible to provide accurate estimation of such data in Table 1. Immediately after the snow thawing (in closed bags), 10 ml of non-filtered aliquot (homogenized by shaking) was acidified with 100 μl of trace element grade HNO_3_ (Merck, Germany) to pH <2 in 15-ml Falcon tubes. By addition of concentrated nitric acid, the sample was preserved and kept frozen until the main analysis. This approach (without filtration procedure) enables to measure environmentally accessible (total) trace metals and metalloid concentration in snow samples. Lab glassware used in this study (bottles, plates) were rigorously cleaned according to the special quality assurance procedure: (i) soaking in 3 M HNO_3_ for 2 days, (ii) rinsing with DDW five times, (iii) drying, and (iv) storing in polyethylene bags. In the present work, the thickness of the snow cover corresponded to the height of snow that was accumulated during a single precipitation event. The in situ snow depth measurements were conducted manually through the use of a polyethylene stick (Table 1). The random sampling of the snow cover depth was performed, including four samples (snow columns) in a line from a small study plot (grid 2 m × 2 m). The results showed relatively small errors within a single experiment. The measured values of the snow cover depth within each plot were compared and given as average value in Table 1.

Total concentrations (dissolved and particulate fraction) of metals/metalloid, Cd, Pb, Cr, Cu, Ni, and As in melted snow samples, were determined in accordance with US EPA method 200.7:2001 by atomic absorption spectrometry (AA-7000 Shimadzu, Japan) with graphite furnace atomization and additionally equipped with ASC-7000 autosampler device. This instrument is commonly used in environmental analyses. Determination of Zn was performed based on flame (FAAS) atomization measurements (AA-7000 Shimadzu, Japan). A palladium matrix modifier Pd(NO_3_)_2_ was used to obtain high analytical sensitivity while quantifying Cd, Pb, and As with GF-AAS. Table 2 summarizes the optimized conditions for metal analyses in this study. Routine analyses of total blanks (for the field case: an open sampling bag was exposed to ambient air in order to examine possible contamination during the sampling procedure; for the lab case: closed bags were filled with DDW, to simulate conditions during snow melting) showed the accuracy below 5 % of MLD for all the analyzed trace elements in snow. Tests of procedural blanks showed no contamination of snow samples during the collection, handling, transport, and storage. The determination of metals by GF-AAS was performed in three replications, and the percentage of RSD did not exceed 5 % (for F-AAS 7 %).

**Table 2:** Conditions of the analytical procedure applied for trace metal/metalloid determination in snow samples

Parameter	Unit	Cu	Cr	Cd	Pb	Ni	As	Zn
		GF-AAS						FAAS
Wavelength	nm	324.8	357.9	228.8	283.3	232.0	193.7	213.9
Slit	nm	0.7	0.2	0.7	0.7	0.2	0.7	0.7
Lamp current	mA	8	12	8	10	12	16	5
Drying	°C	60	60	60	60	60		Acetylene flow (l min^−1^)
		120	120	120	120	120	120	
		250	250	250	250	250	250	
Drying time	s	5, 20, 10	5, 20, 10	3, 20, 10	3, 20, 10	3, 20, 10	20,10	1.4
Ashing	°C	500	800	500	700	800	600	Air flow (l min^−1^)
Ashing time	s	25	25	25	25	25	25	15.0
Atomization	°C	2300	2400	2000	2000	2600	2300	–
Atomization time	s	5	3	3	3	3	5	–
Cleaning	°C	2500	2600	2400	2500	2700	2500	–
Cleaning time	s	2	2	2	2	2	2	–
Injection vol.	μl	20	20	20 + 10 Pd 100 mg l^−1^	20 + 5 Pd 100 mg l^−1^	20	20 + 10 Pd 100 mg l^−1^	–
LOD	ppb	0.03	0.02	0.003	0.03	0.1	0.1	2
LOQ	ppb	0.1	0.06	0.01	0.1	0.03	0.03	6

All lamp modes were BGC-D2

*BGC* background correction, *D2* deuterium lamp

In addition, pH and EC in melted snow samples were determined at 25 °C using a portable multiparameter SevenGo Duo (Mettler Toledo) equipped with automatic temperature compensation system.

### Chemical and meteorological data analysis

Descriptive statistics of TM concentration for each snow measurement was performed using Statistica 10.0 software. Data were checked for normality, outliers, and distribution pattern to apply relevant statistical test for examination of differences in concentrations between snow events. Pearson’s correlation (*p* < 0.05) was used to establish statistically significant dependencies between trace elements measured in the snow cover. A multivariate statistical method principal component analysis (PCA) was used to identify potential TM sources in the snow cover during the whole observation period. Meteorological data, i.e., air temperature (T), atmospheric pressure (p), relative humidity (Rh), and wind speed and direction, were registered simultaneously on the WIOŚ weather station. Moreover, to estimate the impact of macro-regional/regional and distant TM source areas, modeling data retrieved from HYSPLIT NOAA model were analyzed. The 4-day air mass backward trajectory (BT) plots were generated using archive meteorological database GDAS (Draxler and Rolph 2013). Three different altitudes, i.e., 500, 1000, and 1500 m, were examined, roughly corresponding to daily height of the urban planetary boundary layer.

## Results and discussion

### Trace element concentration in snow

Table 3 presents the statistical summary of trace element and pH and EC measurements in a the shallow snow cover collected between January and March 2013 in Poznań. In general, all the measured trace metals showed large variation in mean concentration, between 0.08 μg L^−1^ (Cd) and 4.93 μg L^−1^ (Pb), with a standard deviation of 0.05 and 5.22 μg L^−1^, respectively. The concentration of lead ranged from 0.42 to 34.1 μg L^−1^, and in 90 % of the freshly fallen snow samples, it was found to be between 0.79 and 16.4 μg L^−1^. The overall variation in concentration of Cu, Ni, and Cr was comparable to Pb, however, much higher than As. For arsenic, lower quartile was 0.51 μg L^−1^, whereas 75 % (Q3) of the obtained results showed concentration below 0.88 μg L^−1^. The maximum concentrations for Zn and Ni were similar, 31.0 and 31.4 μg L^−1^, respectively. For Cd, the peak concentration was estimated to be 0.25 μg L^−1^, whereas Q1 was 0.05 μg L^−1^. The average and median Cu values in snow samples were higher than the values observed for As and Cr. All variables in the chemical dataset were normally distributed. However, in several snow episodes, the selected metals (except for Pb) exhibited concentrations below the detection limit. Results of pH varied from a minimum of 3.93 to a maximum of 6.12, with a mean of 4.80 (±0.40). The range of EC measurements was 4.80–79.20 μS cm^−1^ (average of 20.69 ± 14.01 μS cm^−1^), whereas a vast majority of snow samples (90 %) had EC values between 7.07 and 44.9 μS cm^−1^.

**Table 3:** Trace metal concentration and pH and EC values determined in snow samples from the sampling station in Poznań, Poland (2013)

TE	*N*	Mean	SD	Median	Min.	Max.	Skewness	Kurtosis	Q1	Q3	Quartile (5–95)
Cu	83	2.03	2.67	1.00	<MDL	13.7	3	7	0.49	2.24	0.22–8.94
Cd	84	0.08	0.05	0.07	<MDL	0.25	2	3	0.05	0.10	0.02–0.18
Ni	83	3.77	4.88	2.29	<MDL	31.4	4	15	1.37	3.65	0.83–13.9
Pb	84	4.93	5.22	3.19	0.42	34.1	3	12	1.89	5.82	0.79–16.4
Cr	83	0.40	0.46	0.25	<MDL	2.36	3	8	0.16	0.44	0.09–1.61
As	84	0.71	0.31	0.59	<MDL	1.58	1	0	0.51	0.88	0.36–1.22
Zn	84	13.2	4.93	13.0	<MDL	31.0	1	2	9.40	15.0	6.00–22.0
pH	84	4.80	0.40	4.78	3.93	6.12	0	1	4.52	5.00	4.12–5.47
EC	84	20.7	14.0	15.3	4.80	79.2	2	3	10.7	28.1	7.07–44.9

Concentrations are given in micrograms per liter

*TE* trace element, *N* number of samples, *Q1* 25th percentile, *Q3* 75th percentile, *MDL* method detection limit

### Intra-seasonal pattern of trace element concentration in the shallow snow cover

Previous studies showed that trace metals in the urban snow cover undergo numerous processes, and their seasonal variation is controlled by several parameters, i.e., emission source impact, postdepositional processes, and meteorology (Loranger et al. 1996; Cereceda-Balic et al. 2012; Vasić et al. 2012).

**Fig. 2:**
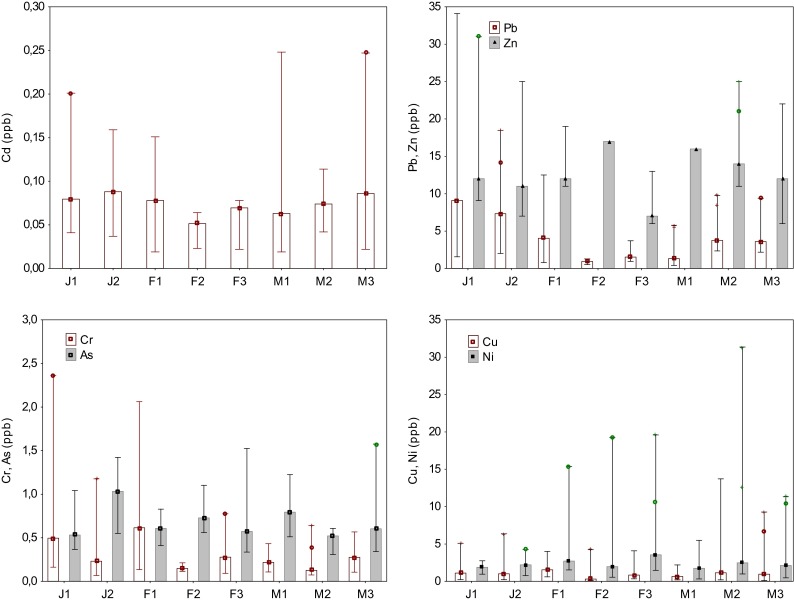
Intra-seasonal variation in trace metal/metalloid concentration in the urban snow cover from Poznań in 2013

Figure 2 illustrates variation of trace element concentrations in the shallow snow cover collected in Poznań during the 3-month field campaigns. In general, maximum concentration of individual species did not occur during the same snow events, suggesting a difference in the conditions of the snow cover formation, industrial activities, air mass transport, and fluctuations in air temperature. High variability in As concentrations was found in March (M1 and M3), whereas the lowest value occurred in February (F1). Intra-seasonal variation in the median As concentration was not statistically significant (Kruskal-Wallis test, *p* < 0.05). Snow samples collected during the J1 experiment exhibited higher concentration of Pb and Zn, although the mean Zn reached maximum during the F2 measurement, as opposite to Pb, indicating the contribution of different emission sources (Fig. 2). On the other hand, these elements were positively correlated (*r* = 0.93, *p* < 0.05) during the whole sampling period. The largest variation in Zn concentrations in snow samples was found between J1 and F2 snow events and might be explained by differences in the prevailing wind direction (N-NE vs. S-SE, respectively) and temperature values (−6.6 vs.−0.4 °C, respectively), as shown in Table 1.

The peak concentration for Cr as well as for Pb was measured during the J1 experiment. The concentrations sharply declined during the J2 snow event. For all the snow measurements in March, Cr concentrations showed less variation compared to data registered in January and February. An opposite trend was found for Ni. In the case of this metal, elevated concentrations were observed during the F1, F2, and F3 snow events, and the maximum value was found at the end of the sampling period (M2). The copper concentration remained relatively constant over the whole observation period, except for two snow episodes in March (M2 and M3) when Cu content in the shallow (25 and 20 cm, respectively) snow cover significantly increased.

### Trace metals in the snow cover—comparison with other locations

A brief synthesis of the available data on trace metal/metalloid contents in various types of snow cover from geographically different sites is given in Table 4. In general, mean Cu concentration in snow samples from Poznań did not exceed 2.03 μg L^−1^ and was 2 orders of magnitude higher than the values recently observed by Bacardit and Camarero (2010) at non-urban sites in Maladeta (Central Pyrenees, Spain). The average Cu value was also much higher than the values determined in snow samples from mountain regions in Italian Alps (0.72 μg L^−1^, Gabrielli et al. 2006); however, it was almost consistent with data from previous studies by Carling et al. (2012) conducted at traffic-impacted site in central Wasatch Mountains (Utah, USA). The highest Cu values in the snow cover were found at the urban expressway site in Montreal, reflecting direct contamination by road activities (Loranger et al. 1996). Notably, several observations of high traffic sites have confirmed that brake abrasion may significantly affect chemical composition of the snow cover (Viklander 1998). For example, Engelhard et al. (2007) found extremely high mean concentration of Cu in urban snow, ca. 630 μg L^−1^. Besides the road traffic and road dust re-suspension, the anthropogenic emission from smelters could be a significant source of copper in the urban atmosphere (Xia and Gao 2011). Cereceda-Balic et al. (2012) identified some industrial activities, i.e., mining and smelting, as dominant Cu sources in snow collected in Cerro Colorado (Chile). These data are in quantitatively good agreement with the results obtained by Loranger et al. (1996).

**Table 4:** Comparison of the mean concentration of total trace elements (μg L^−1^) in the urban snow cover in Poznań and other locations. The “bql” is the amount of trace metal below the quantification limit

Site and data	Type	Cu	Cd	Ni	Pb	Cr	As	Zn	Reference
Poznań, Poland (January–March 2013)	U	2.03	0.08	3.77	4.93	0.40	0.71	13.2	This study
Council, Alaska, USA (March 2002)	M		0.02	0.11	0.60		0.09	0.75	Douglas and Sturm (2004)
Selawik, Alaska, USA (January 2002)	M		0.01	0.62	0.35		0.19	1.71	
Wasatch Mountain, Utah, USA (December 2009–April 2010)	M	3.00	–	0.68	2.30	0.77	0.82	7.66	Carling et al. (2012)
Italian Eastern Alps (December 1997–April 1998)	M	0.72	0.06		1.80	0.10		3.50	Gabrielli et al. (2006)
Maladeta, Spain (March 2005)	M	0.06	bql	0.06	1.92		bql	2.72	Bacardit and Camarero (2010)
Central Greenland (1991–1995)	R	0.004			0.011			0.03	Barbante et al. (2003)
Montreal, Canada (January–February 1993)	U/E	34–51						29.0–143.0	Loranger et al. (1996)
Novy Sad, Serbia (December 2009)	U/C	<4.0			0.4–1.3			<30.0	Vasić et al. (2012)
Innsbruk, Austria (January–February 2006)	U/HT	630	3.87		205			1370	Engelhard et al. (2007)
Cerro Colorado, Chile (October 2003, 2008, 2009)		33.29	0.72	0.70	19.48	0.01	0.55	29.59	Cereceda-Balic et al. (2012)

*U* urban, *M* mountain, *R* remote, *E* expressway, *C* crossroad, *HT* high traffic

It should be noticed that peak concentrations of trace elements in a freshly fallen snow are a combination of numerous factors such as meteorological conditions (decrease in mixing height and wind velocity that mitigates vertical transport and consequently enhances local accumulation of pollutants nearby the emitters) and intensive emission from different types of sources (industrial/commercial). Results from this study clearly showed that multiple anthropogenic sources of Cu affect metal distribution in the shallow snow cover and could confirm strong source-receptor relationships as reported in aforementioned studies. Due to the fact that the sampling site was not strictly influenced by heavy traffic, the exhaust emission of Cu from petrol and diesel vehicles as a predominant source of this metal in the snow cover was probably less visible compared with the data given by Engelhard et al. (2007). Perhaps, high Cu concentration in snow samples measured in Poznań reflects local and regional transport of Cu-enriched particles from various industrial activities such as fossil fuel combustion, metal smelting, metallurgical processes, and road traffic as well. Similar trend was observed for other trace elements analyzed in this study. An increase in energy use for domestic heating, especially in nearby urban residential sector could contribute to the release of a large amount of anthropogenic pollutants within the study domain. Previous studies confirmed that urban conditions, in particular within the residential sector and road traffic, significantly enhance the atmospheric burden of metalliferous pollutants (Siudek et al. 2011; Moreno et al. 2011). A detailed analysis of meteorological situation (both backward trajectory simulations and on-site wind profiles) during the F1, F2, and M2 snow experiments in Poznań showed that air masses advected from S and SE directions, i.e., from highly urbanized zone (Table 1), were frequent.

The most important anthropogenic source of arsenic in the urban atmosphere is non-ferrous metal production, smelting, and steel industry (Sánchez-Rodas et al. 2007). In Poznań, the mean As content in snow samples was 0.71 μg L^−1^, which was similar to the value reported by Carling et al. (2012) at high mountain stations. However, for other mountain regions, this metalloid was observed at relatively low concentration levels as compared to the present study (Table 4). These discrepancies are probably due to different contribution from urban/industrial activities, different characteristics of the observation site, elevation, sampling period, and meteorology.

The snow cover in Poznań was highly enriched in Zn and Pb (13.2 ± 4.93 and 4.93 ± 5.22 μg L^−1^, respectively) compared to other analyzed elements. The mean concentration level of Zn reported in this study was lower than the value measured by Vasić et al. (2012) and Loranger et al. (1996). High Zn amounts in the short-term snow cover in Novy Sad (<30.0 μg L^−1^) and Montreal (29.0–143.0 μg L^−1^) were directly associated with the pollution input from urban emission sources. These sites were located near crossroads and thus affected by oil refining and combustion. In contrast, results from snow measurements at remote sites in Greenland and Alaska showed mean concentrations of Zn below 1.0 μg L^−1^. For example, in Council (USA), the concentration of zinc was 0.75 μg L^−1^ (Douglas and Sturm 2004). In snow samples from Central Greenland, Barbante et al. (2003) found extremely low concentrations of Zn and Pb (0.03 and 0.011 μg L^−1^, respectively). On the contrary, elevated concentrations of Pb were measured in alpine snowpack by Engelhard et al. (2007). In their study, the average content of lead in snow samples equaled 205 μg L^−1^, reflecting both specific meteorological conditions and the predominant role of exhaust emission and re-suspension of Pb-enriched particles at that site. Sakata et al. (2014) found that Pb species in size-fractionated aerosols could occur from a wide spectrum of sources, i.e., fly ash derived from oil combustion. Municipal solid waste incinerations were generally assigned to the fine aerosol fraction, whereas road dust was the main Pb source in coarse particles. Such sources were also identified close to the urban sampling site in Poznań, providing an evidence for the effective accumulation of trace metal-related aerosols of different size in the snow cover.

The Ni concentration measured in urban snow in Poznań was significantly higher than that observed by Douglas and Sturm (2004) in northwestern Alaska (0.11–0.62 μg L^−1^) or by Carling et al. (2012) in Utah (0.68 μg L^−1^), whereas mean levels of Cd were 50 times lower than those found by Engelhard et al. (2007). The wintertime variation in Ni concentration in Poznań could be primarily affected by intensified residential combustion. Previous measurements of heavy metals in urban particulate matter have shown that cadmium is generally associated with fine particles that are more prone to being transported from their original emission sources (Zereini et al. 2005). The shallow snow cover in Poznań obviously had higher content of Cd compared to that from mountain regions, presented in Table 4, where this metal was detected at very low concentration level or bql. Similar trend was observed in terms of Cr; however, the mean concentration was higher in central Wasatch Mountain (0.77 μg L^−1^) than in Poznań (0.40 μg L^−1^), indicating different chemical transformation of this metal in the snow cover of both sites, different geographic position, and proximity to anthropogenic emission sources.

### Identification of TM origin in the snow cover using PCA

In the present study, a principal component analysis (PCA) with varimax normalized algorithm was applied for dataset of nine variables (metal concentrations, pH, and EC values) to identify potential sources of trace elements in the shallow snow cover in Poznań. According to “Kaiser criterion,” only components with eigenvalue above 1 were taken into consideration (Costabile et al. 2008). The PC1, PC2, and PC3 together explained 64 % of the total variance. The results of PCA analysis are displayed in Table 5.

**Table 5:** PCA loadings calculated for nine chemical variables determined in shallow snow samples in Poznań

Variable	Principal component		
	PC1	PC1	PC3
Cu	−0.27	0.11	*0.84*
Cd	−0.34	*0.65*	−0.07
Ni	0.12	−0.12	*0.86*
Pb	0.01	*0.85*	0.10
Cr	0.48	*0.72*	0.10
As	−0.08	0.30	−0.15
Zn	−0.40	*0.59*	0.04
pH	*0.87*	−0.06	−0.11
EC	−*0.88*	0.19	0.01
Eigenvalue	2.56	1.76	1.46
Percent of variance	28	20	16
Cumulative percentage	28	48	64

The statistically significant results of the PCA analysis (>0.5) are given in italic type

The first principal component (PC1) explained 28 % of the overall variance, with two parameters characterized by high loading (>0.8), pH (positive), and EC (negative). This correlation might highlight the role of pH changes in trace element solubility in the snow cover and pre-/postdepositional processes as well. Similar strong relationships between the solubility effect and pH range were previously reported for Zn, Cu, and Cd in rainwater samples (Chester et al. 2000; Başak and Alagha 2010). Furthermore, the variation in pH was also considered as a useful factor while discussing complexation of the analyzed metals.

The second principal component (20 % of total variance) grouped four elements such as Pb, Cr, Cd, and Zn, with loading up to 0.5. The obtained values may indicate mixed sources of snow contamination in Poznań. Consequently, metallurgical processing, electric power plants, refuse incineration (Cd), fossil fuel combustion (Cr), and particularly road traffic including exhaust emission from petrol and diesel vehicles (Pb, Zn, Cd) + tire abrasion, processing of non-ferrous metals, and steel production (Zn) altogether can be considered as dominant sources of trace elements that were emerged from PC2.

The third PC accounted for 16 % of the total variance, with high loading for Cu (0.84) and Ni (0.86). The result suggests strong influence of industrial processes such as iron and steel processing, non-ferrous smelting, waste incineration (Cu), and traffic emission (Ni). These metals can also be co-emitted from fuel burning. In addition, only As (indicator of combustion sources) was not related to any of the principal components.

To summarize, both PC2 and PC3 clearly indicated strong impact of multiple anthropogenic activities on chemical composition of snow in this site. High scores of Pb, Cr, Cu, and Ni could be linked to local/regional air pollutant transport with the contribution from remote industrial areas as well as re-suspension—that will be discussed in the next section.

### Atmospheric long-range transport of TMs

**Fig. 3:**
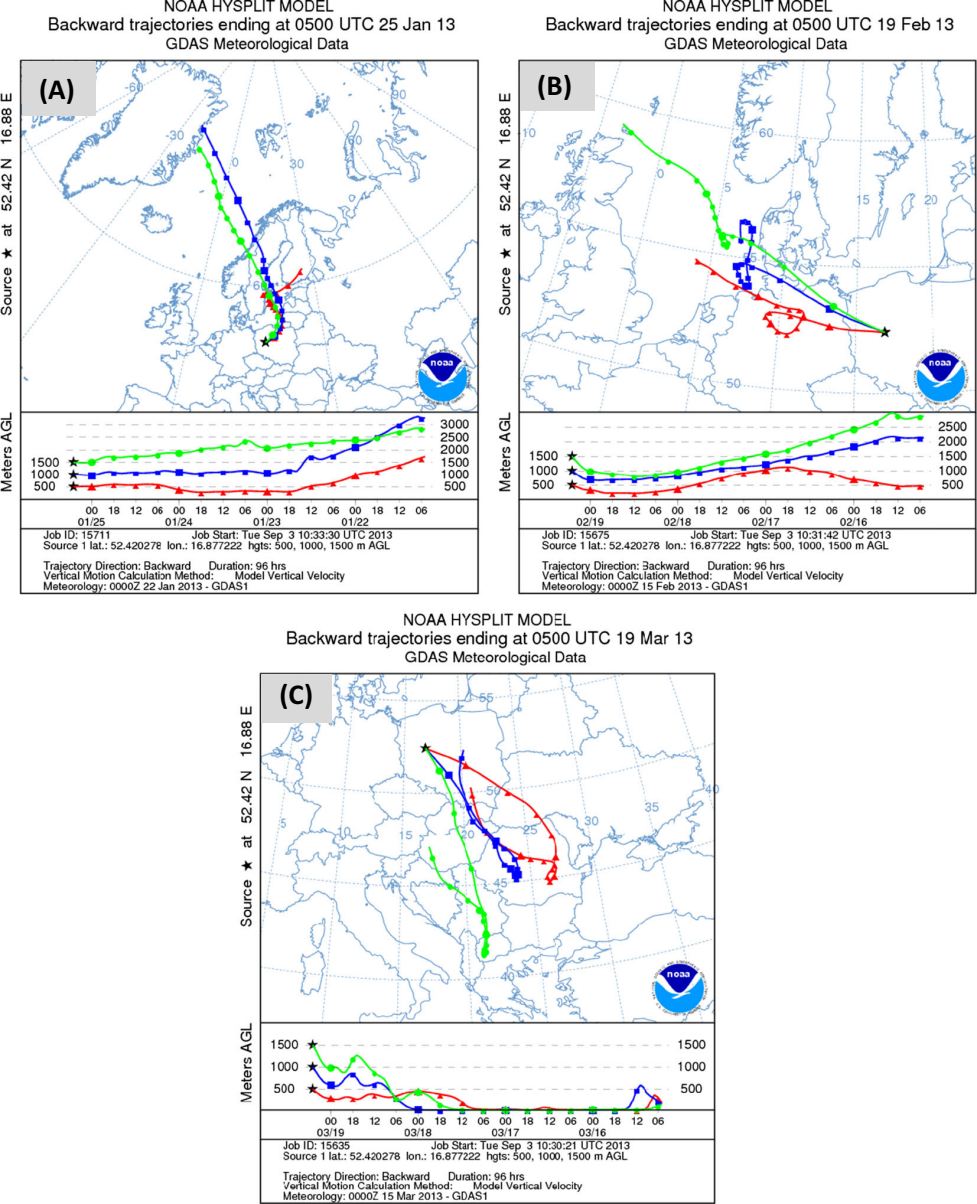
Four-day backward air mass trajectories calculated with HYSPLIT model for snow measurements in Poznań, ending on **a** 25 January 2013, **b** 19 February 2013, and **c** 19 March 2013

In Poland, during the “heating period” (typically between November and March), anthropogenic emission of potentially toxic substances from coal combustion is much higher than during the non-heating season, causing the pronounced increase in air pollution (Siudek et al. 2011). Previous observations confirmed that local atmospheric input of TMs might be significantly enhanced by the transport of polluted air masses from distant source areas (Gabrielli et al. 2008). In order to examine the source-based anthropogenic impact attributed to both local/regional and long-range transport and estimate the influence of factors controlling the seasonality of TMs in the snow cover, different groups of meteorological situations with significantly higher TM contents in the snow cover were considered. Due to the fact that backward trajectory analysis showed various types of air masses that were passing over Poznań at variable elevation heights during the sampling days, three representative examples that met aforementioned criteria were examined (Fig. 3). Such an approach also allowed to find other distant TM sources that affect chemical composition of the snow cover in Poznań.

First, snow episode with significantly higher concentrations of Pb, Zn Cr, and Cd was observed on 25 January (Fig. 3a). The 96-h backward trajectory (BT) simulation for that high pollution event showed the predominance of northerly and northeasterly circulation. The BT results revealed some potential source areas for TMs accumulated in the snow cover at Poznań urban site, i.e., northern high latitudes (Greenland, North Atlantic), Scandinavia region, Russia, Lithuania, and northeastern Poland. Due to the fact that urban/industrial emission in polar regions and northern Scandinavia is rather low, these regions had probably minor contribution to TMs in the snow cover in Poznań (EMEP 2013). In this way, chemical composition of the snow cover was more effectively influenced by distribution of pollution tracers from “industrial hot spots” located around the eastern part of the Baltic Sea drainage basin (Aas and Breivik 2013). In addition, the parallel anemometric observations pointed to relatively low wind velocity (ranging from 0.1 to 1.1 m s^−1^), suggesting transport of pollutants from local and regional anthropogenic sources, such as residential boilers, fossil fuel combustion, military area in Biedrusko, and waste incineration, toward the sampling site. Moreover, the favorable atmospheric conditions (nocturnal inversion layer) might lead to the accumulation and higher concentrations of TMs in the lower atmosphere over the study region just before the J1 snow episode. The air temperature during those days was also very low (ranging from −12.4 to −0.2 °C), causing the intensified residential fuel consumption. The concentrations of Zn and Pb in the snow cover sampled during the J1 experiment was significantly higher compared to other measurements. Both metals could be co-emitted from traffic-related sources at urban and sub-urban settings as described by Councell et al. (2004). Due to the limited number of data presented in this study, it cannot be clearly indicated that transportation activities (i.e., daily traffic volume) were the main contributor of Zn to the snow cover and that they control its concentration levels.

In contrast, during the snow experiment on 19 February (F3), the air temperature varied between −1.2 and 1.6 °C, whereas the speed of wind slightly increased from 1.1 to 3.7 m s^−1^ (Table 1). Figure 3b shows that during this experiment, high frequency of northwest to west advection was observed in the examined region. The elevated concentrations of As and Ni were probably caused by the enhanced emission from local combustion processes and/or additional input from long-range atmospheric transport. For comparison, the same transport pattern was observed during the J2 experiment (not shown). However, the peak concentration of Zn, Cd, Cr, Cu, and Pb was much higher compared to the results obtained in F3 but still lower than those found in other sites (Engelhard et al. 2007). One should be noted that the differences observed in TM content in the snow cover in Poznań were caused not only by seasonal variation in pollutant concentration (emission from residential and commercial sector) but also by a combination of chemical and atmospheric conditions. It is well known that low temperature together with the increase in humidity largely affects TM transformational/depositional processes in the lower atmosphere including coagulation and sorption of compounds onto airborne particles. Furthermore, this temperature-induced mechanism has fundamental meaning while considering partitioning between soluble and particulate phase.

Finally, the elevated concentrations of Ni, Zn, and Cu were measured in the snow cover collected during the M2 observations. In this case, both Ni and Cu concentrations in snow cover were almost twofold higher than those measured during the other snow campaigns. Ni is identified as a source marker for fossil fuel combustion, high-temperature metallurgical processes, stainless steel production, chemical factories, food processing industry, municipal waste incineration, and smelting (Gregurek et al. 1998). Anthropogenic sources of Zn in the atmosphere are mostly related to metal production, waste incineration, combustion processes, with minor role of phosphate fertilizers, and cement production. Similar urban/industrial sources of the metals were identified within our study domain. Therefore, it can be concluded that local combustion processes could be the dominant source of these metals in the shallow snow cover. The similarities in the origin of metals during the M3 experiment were also supported by the results of PCA analysis for Ni (0.86) and Cu (0.84), as shown in Table 3.

Based on the backward trajectory calculation for the M2 event, it was demonstrated that the sampling site was under the permanent influence of air masses originated from central and southeastern Europe (Hungary, Serbia, Slovakia, Romania, Ukraine). This large region could have a significant contribution to snow contamination during the March experiment presented in Fig. 3c. The southern part of Poland (Upper Silesia) was particularly expected to become a significantly important source area of TMs in the snow cover due to large dust emission (32 800 t) estimated on 22 % of the country-based annual mean value (Smołka-Danielowska 2006). This region (ca. 300 km from the sampling site) is densely buildup with various kinds of industrial sources and thus likely to have a large-scale impact on the atmosphere. Similar features were documented previously (Siudek et al. 2011).

## Conclusions

Total trace metal concentrations were quantitatively determined in a shallow snow cover from an urban region in central Poland during the winter season of 2013. A newly validated sampling method was applied to observe the dynamics in distribution of chemical species and to determine how meteorological conditions contribute to TM variation during the snowfall events. The chemical composition of the snow cover showed relatively high intra-seasonal variability in total TM content during the whole snow season, as a combination of changes in weather conditions (fluctuation of temperature and relative humidity) and the contribution of local/regional anthropogenic emission sources. Maximum trace element concentrations (μg L^−1^) were ranked as follows: Pb (34.90) > Ni (31.37) > Zn (31.00) > Cu (13.71) > Cr (2.36) > As (1.58) > Cd (0.25).

Large differences in Zn concentration reflected the significant influence of industrial activities related to metal production, waste incineration, and high-temperature processes. Concentration levels of Pb and Ni were lower as compared with values determined over largely polluted areas, indicating important influence of anthropogenic sources in nearby area (i.e., traffic, fossil fuel combustion, industrial processes, re-suspension of soil/dust,) on the contamination of the sampling site.

Detailed BT analysis provided an evidence that long-range transport could significantly affect the chemical composition of the snow cover collected in Poznań. For example, the snow cover with extremely high Pb, Zn, and Cr amounts was observed during northerly and northeasterly flow of polluted air masses, while for Ni, the elevated concentrations were found during the southern and southeastern advection.

The results presented in this study are of fundamental importance for understanding the transformation pattern of impurities in the snow cover in urban regions. The fact that more than 80 % of the analyzed snow samples were characterized by high amount of Pb, Ni, Zn, and Cr suggests the significant contribution of local and regional urban/industrial activities.

## References

[CR1] Aas, W., & Breivik, K. (2013). *Heavy metals and POP measurements*. EMEP/CCC-Report 4/2013, Kjeller.

[CR2] Bacardit M, Camarero L (2009). Fluxes of Al, Fe, Ti, Mn, Pb, Cd, Zn, Ni, Cu and As in monthly bulk deposition over the Pyrenees (SW Europe): the influence of meteorology on the atmospheric component of trace element cycles and its implications for high mountain lakes. Journal of Geophysical Research – Biogeosciences.

[CR3] Bacardit M, Camarero L (2010). Atmospherically deposited major and trace elements in the winter snowpack along a gradient of altitude in the Central Pyrenees: the seasonal record of long-range fluxes over SW Europe. Atmospheric Environment.

[CR4] Barbante C, Boutron C, Morel C, Ferrari C, Jaffrezo JL, Cozzi G, Gasparia V, Cescona P (2003). Seasonal variations of heavy metals in central Greenland snow deposited from 1991 to 1995. Journal of Environmental Monitoring.

[CR5] Başak B, Alagha O (2010). Trace metals solubility in rainwater: evaluation of rainwater quality at a watershed area, Istanbul. Environmental Monitoring and Assessment.

[CR6] Becagli S, Sferlazzo DM, Pace G, di Sarra A, Bommarito C, Calzolai G, Ghedini C, Lucarelli F, Meloni D, Monteleone F, Severi M, Traversi R, Udisti R (2012). Evidence for heavy fuel oil combustion aerosols from chemical analyses at the island of Lampedusa: a possible large role of ships emissions in the Mediterranean. Atmospheric Chemistry and Physics.

[CR7] Carling GT, Fernandez DP, Johnson WP (2012). Dust-mediated loading of trace and major elements to Wasatch Mountain snowpack. Science of the Total Environment.

[CR8] Cereceda-Balic F, Palomo-Marin MR, Bernalte E, Vidal V, Christie J, Fadic X, Guevara JL, Miro C, Pinilla G (2012). Impact of Santiago de Chile urban atmospheric pollution on anthropogenic trace elements enrichment in snow precipitation at Cerro Colorado, Central Andes. Atmospheric Environment.

[CR9] Chester R, Nimmo M, Fones GR, Keyse S, Zhang J (2000). The solubility of Pb in coastal marine rainwaters: pH-dependent relationships. Atmospheric Environment.

[CR10] Costabile F, Birmili W, Klose S, Tuch T, Wehner B, Wiedensholer A, Franck U, König K, Sonntag A (2008). Spatio-temporal variability and principal components of the particle number size distribution in an urban atmosphere. Atmospheric Chemistry and Physics.

[CR11] Councell TB, Ducjenfield KU, Landa ER, Callender E (2004). Tire-wear particles as source of zinc to the environment. Environmental Science and Technology.

[CR12] Dossi C, Ciceri E, Giussani B, Pozzi A, Galgaro A, Viero A, Viagano A (2007). Water and snow chemistry of main ions and trace elements in the Karst system of Monte Pelmo massif (Dolomites, Eastern Alps, Italy). Marine and Freshwater Research.

[CR13] Douglas TA, Sturm M (2004). Arctic haze, mercury and the chemical composition of snow across northwestern Alaska. Atmospheric Environment.

[CR14] Draxler, R.R., & Rolph, G.D. (2013). HYSPLIT (HYbrid Single-Particle Lagrangian Integrated Trajectory) Model access via NOAA ARL READY Website http://www.arl.noaa.gov/HYSPLIT.php). College Park: NOAA Air Resources Laboratory.

[CR15] EMEP (2013). Atmospheric supply of nitrogen, lead, cadmium, mercury and dioxin/furans to the Baltic Sea in 2011. EMEP/MSC-W TECHNICAL REPORT 2/2013, Oslo.

[CR16] Engelhard C, De Toffol S, Lek I, Rauch W, Dallinger R (2007). Environmental impacts of urban management—the alpine case study of Innsbruck. Science of the Total Environment.

[CR17] Gabrielli P, Cozzi G, Torcini S, Cescon P, Barbante C (2006). Source and origin of atmospheric trace elements entrapped in winter snow of the Italian Eastern Alps. Atmospheric Chemistry and Physics Discussions.

[CR18] Gabrielli P, Cozzi G, Torcini S, Cescon P, Barbarante C (2008). Trace elements in winter snow of the dolomites (Italy): a statistical study of natural and anthropogenic contributions. Chemosphere.

[CR19] Gregurek D, Reimann C, Stump EF (1998). Trace elements and precious metals in snow samples from the immediate vicinity of nickel processing plants, Kola Peninsula, northwest Russia. Environmental Pollution.

[CR20] Loranger S, Tétrault M, Kennedy G, Zayed J (1996). Manganese and other trace elements in urban snow near an expressway. Environmental Pollution.

[CR21] Melaku S, Morris V, Raghavan D, Hosten C (2008). Seasonal variation of heavy metals in ambient air and precipitation at a single site in Washington, DC. Environmental Pollution.

[CR22] Moreno, T., Querol, X., Alastuey, A., Reche, C., Cusack, M., Amato, F., Pandolfi, M., Pey, J., Richard, A., Prevot, A. S. H., Furger, M., & Gibbsons, W. (2011). Variations in time and space of trace metals aerosol concentrations in urban areas and their surroundings. *Atmospheric Chemistry and Physics, 11*, 9415–9430.

[CR23] Pacyna JM, Pacyna EG (2001). An assessment of global and regional emission of trace metals to the atmosphere from anthropogenic sources worldwide. Environmental Review.

[CR24] Sakata K, Sakaguchi A, Tanimizu M, Takaku Y, Yokoyama Y, Takahashi Y (2014). Identification of sources of lead in the atmosphere by chemical speciation using X-ray absorption near-edge structure (XANES) spectroscopy. Journal of Environmental Sciences.

[CR25] Sánchez-Rodas D, Sánchez de la Campa AM, de le Rosa JD, Oliveira V, Gómez-Ariza JL, Querol X, Alastuey A (2007). Arsenic concentration of particulate matter (PM10) in an industrialized urban site in southwestern Spain. Chemosphere.

[CR26] Siudek P, Falkowska L, Urba A (2011). Temporal variability of particulate mercury in the air over the urbanized zone of the southern Baltic. Atmospheric Pollution Research.

[CR27] Siudek P, Falkowska L, Frankowski M, Siepak J (2014). An investigation of atmospheric mercury accumulated in the snow cover from the urbanized coastal zone of the Baltic Sea, Poland. Atmospheric Environment.

[CR28] Smołka-Danielowska D (2006). Heavy metals in fly ash from a coal-fired power station in Poland. Polish Journal of Environmental Studies.

[CR29] Vasić MV, Mihailović A, Kozmidis-Luburić U, Nemes T, Ninkov J, Zeremski-Škorić T, Antić B (2012). Metal contamination of short-term snow cover near urban crossroad: correlation analysis of metal content and fine particles distribution. Chemosphere.

[CR30] Viklander, M. (1998). Snow quality in the city of Lulea, Sweden: time-variation of lead, zinc, copper and phosphorus. *Science of the Total Environment,1–2*, 103–112. 10.1016/s0048-9697(98)00148-x 9618929

[CR31] Wiener JG, Krabbenhoft DP, Heinz GH, Scheuhammer AM, Hoffman DJ, Rattner BA, Burton GA, Cairns JS (2003). Ecotoxicology of mercury. Handbook of ecotoxicology.

[CR32] Xia L, Gao Y (2011). Characterization of trace elements in PM2.5 aerosols in the vicinity of highways in northeast New Jersey in U.S. east coast. Atmospheric Pollution Research.

[CR33] Zereini F, Alt F, Messerschmidt J, Wiseman C, Feldmann I, von Bohlen A, Müller J, Liebl K, Püttmann W (2005). Concentration and distribution of heavy metals in urban airborne particulate matter in Frankfurt am Main, Germany. Environmental Science and Technology.

